# Mumbod? A comparison of body image and dietary restraint among women with younger, older, and no children

**DOI:** 10.1177/1359105320967422

**Published:** 2020-10-28

**Authors:** Zali Yager, Ivanka Prichard, Laura Hart, Stephanie R Damiano

**Affiliations:** 1 Victoria University, Australia; 2 Flinders University, Australia; 3 La Trobe University, Australia; 4 University of Melbourne, Australia

**Keywords:** body image, maternal mental health, mother, objectification, postpartum

## Abstract

Pregnancy and the postpartum period are times of significant body, and body image changes for women. Adult women (*N* = 885) aged 21 to 47 years old completed an online questionnaire. Mothers of young children (0–5 years) had significantly lower levels of body shame, self-objectification, and dietary restraint than women without children, and women with school-aged (6–10 years) children had significantly lower self objectification than women without children, once we controlled for age. BMI predicted body shame more than motherhood status. This research has implications for the development of appropriate body image interventions for adult women.

## Introduction

Body dissatisfaction among women is so prevalent that the phenomenon has been described as “normative discontent” for over 30 years (Rodin et al., 1985). More than one quarter (26.3%) of women are said to be somewhat or extremely dissatisfied with their body (Becker et al., 2017), and 51.1% of women have moderate or marked shape and weight concerns (Becker et al., 2017), with higher prevalence among women with a high body mass index (BMI) (Runfola et al., 2013). In addition, 91% of all women indicate an ideal figure that is smaller than their current figure using silhouette discrepancy measures (Runfola et al., 2013). Overall, body dissatisfaction in women is considered to be problematic yet “relatively stable across the lifespan” (Becker et al., 2017; Runfola et al., 2013; Tiggemann, 2004). Recent German research has confirmed that body dissatisfaction is unaffected by age in women, but that body appreciation improves as women get older (Quittkat et al., 2019).

Body appreciation and positive body image refer to a respect, love, and gratitude for the body, and what it can do (Tylka and Wood-Barcalow, 2015b). Body appreciation is not just the opposite of body dissatisfaction, but a distinct construct that predicts unique variance in self-esteem and intuitive eating (Tylka and Wood-Barcalow, 2015b). Very little prevalence data relating to body appreciation exists, but appreciation is known to increase with age, and women aged over 60 are known to have the highest levels of body appreciation (Becker et al., 2017; Quittkat et al., 2019; Tiggemann and McCourt, 2013). Women with higher levels of body appreciation report higher quality of life (Nayir et al., 2016), and engaging in more positive health behaviors such as physical activity, intuitive eating, and self-care (Tylka and Wood-Barcalow, 2015a), as well as cancer screening and lower alcohol intake (Andrew et al., 2016).

Self-objectification, internalization of cultural appearance ideals, and body shame, are all constructs that are related to both body dissatisfaction and appreciation. Objectification Theory (Frederickson and Roberts, 1997) proposes that the body image of girls and women depends largely on how they think others see and judge them. Within Western culture in particular, women are often conditioned to evaluate themselves based on their physical appearance rather than what their body can do, and in turn treat their body as an object that can and should be manipulated to seek the approval of others (Frederickson and Roberts, 1997). This process is termed self-objectification. Related to this, internalization of appearance ideals refers to the degree to which women have adopted, and believe in the value and importance of, living up to the appearance ideals that are portrayed in their culture. If women have high levels of self-objectification, and high levels of internalization, but they do not believe that their appearance meets the cultural standards of beauty, then they are likely to experience body dissatisfaction and shame. Body shame evolves from a feeling of failure to meet the beauty standards that are prescribed, and like shame in general, tends to result in feelings of “I am a bad person,” rather than the feelings of guilt that lead to “I did something bad” (Noll and Fredrickson, 1998). Body shame has been found to mediate the relationship between BMI and self-esteem (Pila et al., 2015). Further, if women believe that their body is larger than the societal “ideals,” and have high levels of body dissatisfaction and shame, then they are more likely to engage in unhealthy dietary restraint to modify their weight and shape (Dakanalis et al., 2015).

A great deal of research indicates that pregnancy and the postpartum period are a time of significant identity, body, and body image changes for women (Rallis et al., 2007). In fact, the extent of the hormonal, physical, and identity changes that occur throughout pregnancy and the postpartum period are akin to puberty, and described using the term “matrescence,” coined by anthropologist Dana Raphael in the 1970’s (Raphael, 1973). Matrescence describes the complete transformation to becoming a mother—a woman will never actually return to who she was, or what she looked like before having children (Taylor-Kabbaz, 2019). Body dissatisfaction seems to be heightened in early pregnancy, decreases in the third trimester of pregnancy, and rises again in the year after birth—peaking at around 6 to 9 months postpartum (Chan et al., 2020; Rallis et al., 2007). Recent research using network analysis found that women who were pregnant had similar levels of overall body dissatisfaction as non-pregnant women, but pregnant women were more likely to be more dissatisfied with their weight, muscle size, skin tone, fluid retention, energy levels, and overall functioning, but were less dissatisfied with their hips, chest, shoulders, calves, and facial complexion than non-pregnant women (Fuller-Tyszkiewicz et al., 2020). At 6-weeks postpartum, 77.3% of women in a large Chinese study were worried about not being able to return to their pre-pregnancy body shape (Chan et al., 2020). A study conducted among US women who had given birth in the past year found that 68% had an unrealistic goal weight that was lower than their self-reported pre-pregnancy weight by an average of 5.4 kilograms or 11.9 pounds (Lovering et al., 2018). Many quality studies conducted around the world have also reported a relationship between body dissatisfaction and postpartum depression (Chan et al., 2020; Clark et al., 2009; Riquin et al., 2019).

Body appreciation during the pregnancy and postpartum period has not yet been researched as thoroughly as body dissatisfaction. Qualitative work indicates that some women experience higher body appreciation after experiencing their body give birth and engaging in breastfeeding, however some women do not (Raspovic et al., 2020). One study of US women with a child 0 to 12 months old found that women who expected to be able to breastfeed exclusively, but were not able too, experienced lower appreciation of body functionality (Rosenbaum et al., 2020). Mothers with low levels of self-compassion were found to have the lowest levels of body appreciation, and highest levels of depression (Rosenbaum et al., 2020).

The influences on body image in the postpartum period are thought to be predominantly sociocultural. Research has confirmed that the tripartite model of body dissatisfaction fits postpartum women’s experience of their bodies, in that pressure from the media, partner, and peers strengthens the internalization of the thin ideal and thereby increases body dissatisfaction during this time (Lovering et al., 2018). While research has begun to investigate the extent of body appreciation and body dissatisfaction among women across the lifespan, very little work exists to indicate the extent of the differences in body image among women who have, and have not, had children.

This research is important because of the potential psychological and physical health implications for women and mothers, but also their children and others around them. A large volume of research exists demonstrating the relationship between mothers’ and children’s body image, eating attitudes and behaviors, and weight bias (Bergmeier et al., 2020; Damiano et al., 2015; Lease et al., 2016; McBride et al., 2017; Spiel et al., 2012). We suggest that mothers may contribute to the creation of a home environment that is either supportive and body positive, or one that encourages body dissatisfaction, dieting, and objectification, and that at least part of this is related to a mother’s own body appreciation or dissatisfaction. It is therefore important to understand the impact of motherhood, and how this might affect body image, to be able to intervene with resources that might assist mothers and their children.

The aim of this study was to better understand the effects of motherhood on women’s body image. The objective was to examine whether differences exist on measures of body appreciation, body shame, objectification, internalization of appearance ideals, and dietary restraint, between women without children, women with younger children (youngest child aged between 0 and 5 years at the time of the survey), and women with older children (youngest child aged between 6 and 10 years at the time of the survey). The constructs under examination were chosen because they represent interrelated aspects of body image and eating (i.e. dieting) behavior. The age categories were adopted to broadly capture the differences between women who have given birth more recently and are in the more intense pre-school parenting period, compared to those with school-aged children, and to women without children. Given previous research on the links between BMI and body dissatisfaction (Bucchianeri et al., 2013), we also examined the moderating role of BMI. It was hypothesized that:

1. Compared to women without children, women with children (0–10 years) would have lower levels of body appreciation, and poorer body image, as indicated by higher levels of body shame, objectification, internalization of the thin ideal, and dietary restraint. Because their postpartum bodies are still recovering from the pregnancy and birth process, and might be further from the societal “thin ideal,” these women may therefore may desire weight loss in order to get their “pre-baby body” back.
2. Women whose youngest child is aged 0 to 5 years would have poorer body image than women whose youngest child is aged 6 to 10 years, because the effects of pregnancy and birth are more recent.

## Materials and methods

Human Ethics Approval to conduct the study was obtained through [Flinders University Human Ethics Committee, approval number 7481], and mirror approval was sought at Victoria University. Recruitment was conducted online through social media (Facebook, Twitter), using a snowball sampling method from July to September, 2017. This study was conducted as a part of a larger project to explore body image among adult women, and the impact of the documentary film “Embrace.” The Body Image Movement [BIM] posted the link to the study on their Facebook page (251,264 followers as at January 2018), asking for those who have, and have not seen the film Embrace to please complete an online questionnaire, using the software Qualtrics. Participants were also asked to tag their friends in the post, to invite others in their network to participate in the research, whether these other women were members of the BIM or not. Participants completion of the questionnaire was accepted as their consent to participate in this research.

### Participants

Data were provided by 2067 women via the online questionnaire. Due to extensive missing data, 658 participants were excluded from the analyses. Extensive missing data was defined as cases whereby (1) Only 5% or less of the items were completed, or (2) Less than three of the five key outcome measures were completed. Women without children who fell outside the 21 to 47 years age range were also excluded from analysis (*n* = 388), as were women more than 10 years postpartum (*n* = 136), as we were interested in comparing mothers of a similar age group in the intense early years of motherhood. Mann Whitney U tests indicated no significant differences in education or country of birth between participants excluded from the analysis and those included in the analysis (*p* > 0.05). The following analyses were conducted on the final sample size of 885 women.

Three comparison groups were established: (1) Women without children (non-mothers), *n* = 430; (2) Women with young children—less than 5 years postpartum (youngest child is aged 5 years or less), *n* = 281; (3) Women with older children who are 6 to 10 years postpartum (youngest child is aged greater than or equal to 6, and less than or equal to 10 years of age) *n* = 174.

### Measures

The online Qualtrics questionnaire asked about demographics (age, ethnicity, postcode/zipcode, education level, gender, height, and weight), about seeing the film Embrace, and about biological children (how many if any, their age and gender). Participants also completed the following standardized scales to measure body appreciation, body shame, objectification, internalization, and dietary restraint.

#### Body appreciation

The 10-item Body Appreciation Scale-2 [BAS-2] (Tylka and Wood-Barcalow, 2015a) assessed participants’ acceptance of, positive attitudes toward, and respect for their bodies. It uses a 5-point response scale (never-always), with higher scores indicating higher levels of body appreciation (mean scores ranged from 1 to 5). The BAS-2 indicated high internal consistency within the current sample (α = .96)

#### Body shame

Body shame was measured using the Body Shame subscale from the Objectified Body Consciousness Questionnaire (McKinley and Hyde, 1996). This 8-item subscale asks how participants feel about themselves in relation to their weight and appearance. Participant responses are via a 7-point scale (strongly disagree-strongly agree) with higher scores indicating higher levels of body shame (McKinley, 1998). In the current sample, the measure indicated acceptable reliability (α = .68).

#### Self-objectification

The Self-Objectification Questionnaire (Noll and Fredrickson, 1998) asks participants to rank a list of five appearance-based (e.g. sex appeal, weight) and five competence-based (e.g. energy level, physical fitness) body attributes according to how important each of them are to an individual’s physical self-concept. Scores were computed according to the original author’s instructions by summing the ranks for the appearance and competence-based attributes, respectively, and then creating a difference score, so scores range from −25 to 25 (Noll and Fredrickson, 1998), with higher scores indicating higher levels of self-objectification (Noll and Fredrickson, 1998). In order to meet the requirements of the statistical procedures (only numbers greater than zero permitted), the self-objectification scores were shifted upwards by 25 so that scores ranged from 0 to 50 with *higher* scores indicating greater self-objectification.

#### Internalization

Internalization of appearance ideals was measured using the Ideal-body Stereotyping—Revised scale (Stice and Agras, 1998). This six-item measure asks participants to indicate the extent to which they agree that certain female body types/shapes are attractive on a 5-point scale from strongly disagree to strongly agree. High scores indicate higher levels of internalization of the thin ideal. Good internal consistency was observed for this measure in the present study (α = .87).

#### Dietary restraint

Dieting behavior was assessed using the 10-item Dietary Restraint subscale of Dutch Eating Behavior Questionnaire (Van Strien et al., 1986). Participants respond to items on a 5-point scale (never-very often). Higher scores indicate higher levels of dietary restraint. The measure indicated high internal consistency in the current study sample (α = .93).

### Data analyses

All data were analyzed in SPSS version 24. Data were cleaned and manually screened for duplicates using IP address and corresponding demographic data.

A series of ANCOVAs (controlling for age) were used to investigate differences across the three comparison groups on the measures of body image and dietary restraint. Moderation was assessed using hierarchical regression.

## Results

### Demographic characteristics

The following analyses were conducted on the final sample size of 885 women. Women were aged on average 35.29 years (*SD* = 6.51). A total of 281 (31.8%) women were mothers whose youngest child was aged five years or less, 174 (19.7%) were mothers whose youngest child was aged to 10 years. The remaining 430 women (48.6%) did not have children. Women with young children had a mean age of 35.22 (*SD* = 4.77) and mean BMI of 28.18 (*SD* = 7.18). Women with older children had a mean age of 40.83 (*SD* = 3.66) and BMI of 28.76 (*SD* = 6.87), while women with no children had a mean age of 33.10 (*SD* = 7.05) and BMI of 28.51 (*SD* = 8.13). There was a significant difference in the age of women across groups, *F*(2, 882) = 108.81, *p* < 0.001, whereby age was significantly different across all three groups, so we controlled for age in subsequent analyses. There were no significant differences between the three groups according to BMI, *F*(2, 780) = 0.286, *p* = 0.751. Most women were Caucasian or European (*n* = 798, 94.2%), 1.9% Hispanic, 0.3% Aboriginal or Torres Strait Islander, and 1.0% as Asian. There was a significant difference in the proportion of participants in each group who were Caucasian (*X*^2^ = 6.82, *p* = 0.033), as there was a higher proportion of women with no children who were Caucasian (47.24%, *n* = 377) compared to women with young children (33.0%, *n* = 263), and women with school-aged children (19.80%, *n* = 158). The sample was highly educated with the majority holding a Bachelor degree (44.1%) or Master’s degree (20.9%). In addition, 12.9% had completed high school, 2.5% did not complete high school, 4.3% had a doctorate and 15.4% responded “other.” There were no significant differences between the groups on level of education (*p* > 0.05).

### Impact of motherhood on body image

The average scores on all outcome measures are presented in Table 1 (controlling for age). After controlling for the effects of age, there were significant differences across the three groups on body shame, self-objectification, and dietary restraint (see Table 1). Specifically, pairwise comparisons indicated that mothers with younger children (0–5 years) had significantly lower body shame (*p* = 0.006), lower dietary restraint (*p* = 0.003), and lower self-objectification (*p* = 0.009) than women with no children. Women with children aged 6 to 10 years also had significantly lower self-objectification than women who had no children (*p* = 0.050). There were no other significant differences between groups.

**Table 1.** Adjusted means (and standard errors) and differences across groups on body image variables controlling for age.

	Women with young children <5 years	Women with older children 6–10 years	Women with no children	Differences between groups	
	*M_adj_* (*SE*)	*M_adj_* (*SE*)	*M_adj_* (*SE*)	*F*	*p*
Body appreciation	3.39 (.051)	3.41 (.070)	3.26 (.042)	2.655	0.071
Body shame	3.65 (.088)^a^	3.90 (.121)	3.96 (.073)^b^	3.963	0.019
Self-objectification^	12.47 (.745)^a^	12.57 (1.023)^a^	15.02 (.623)^b^	4.047	0.018
Dietary restraint	2.55 (.052)^a^	2.70 (.072)	2.75 (.044)^b^	4.452	0.012
Internalization	2.98 (.050)	3.12 (.069)	3.03 (.042)	1.356	0.258

^ Higher scores indicate higher levels of self-objectification; different superscripts

^a^ and ^b^ denote where significant differences lie, ie., group a is significantly different from group b.

To establish the potential moderating effects of BMI, a series of hierarchical regressions were conducted to investigate the impact of BMI on the relationship between motherhood status and the body image variables (body appreciation, body shame, self-objectification, internalization of the thin ideal). BMI was centered, and two dummy variables were computed for motherhood status: dummy 1 (1 if a mother of a child 0–5 years, 0 otherwise) and dummy 2 (1 if a mother of a child 6–10 years, 0 otherwise). Two product variables were then created by multiplying both dummy variables with centered BMI. Age was entered as a covariate in Step 1 for all regression models. At Step 2, centered BMI and the motherhood status dummy variables were entered. This was followed by the two-way product variables at Step 3. As can be seen from Table 2, there were no significant interaction effects at Step 3 indicating no moderation. However, there were main effects of BMI indicating that BMI was a negative predictor of body appreciation and a positive predictor of body shame. Age was also a significant negative predictor for self-objectification.

**Table 2.** Hierarchical multiple regression analysis predicting body image variables from motherhood status and BMI.

Predictor	Body appreciation		Body shame		Self-objectification		Internalization	
	Δ*R*^2^	*β*	Δ*R*^2^	*β*	Δ*R*^2^	*β*	Δ*R*^2^	*β*
Step 1	.000		.001		.011**		.001	
Age		–.019		.033		–.104**		.030
Step 2	.046***		.046***		.005		.008	
Dummy 1		.033		–.052		–.068		–.030
Dummy 2		.026		.018		–.054		.081
BMI		–.211***		.208***		–.021		–.019
Step 3	.003		.007^a^		.001		.001	
BMI × Dummy 1		–.055		.068		.034		–.015
BMI × Dummy 2		.011		–.055		.013		–.034
Total *R*^2^	.048***		.047***		.017*		.010	
*n*	779		754		765		779	

Dummy 1 = 1 if a mother of a child 0–5 years, 0 otherwise; Dummy 2 (1 if a mother of a child 6–10 years, 0 otherwise).

*** *p* < 0.001, **p < 0.01, *p < 0.05, ^a^.053.

## Discussion

This research sought to investigate whether women with children, and in particular women with young children, have increased body image concerns compared to women without children. Theoretically, women with young children who have recently gone through the bodily and identity changes associated with pregnancy, birth, and breastfeeding in early motherhood are likely to be at a higher weight and further from societal ideals, so we hypothesized that they would experience higher levels of self-objectification, body shame, internalization of appearance ideals, and dietary restraint, as well as lower levels of body appreciation. However, we found that this was not the case. After controlling for age, women with young children were actually found to have significantly lower levels of body shame, self-objectification and dietary restraint than women without children. Women with 6 to 10 year old children also had significantly lower self-objectification than women without children. It therefore seems that motherhood status might be protective of body image and dieting attitudes and behaviors.

There are a few explanations for the relatively lower levels of body shame, objectification, and dietary restraint observed among mothers of young children. First, it may be that during the early phase of motherhood and the postpartum period, women might still consider themselves to be temporarily excused from the need to adhere to societal appearance ideals. Some women report renewed functional purpose, and appreciation of what their body can do during the postpartum period, which may alleviate feelings of body shame (Raspovic et al., 2020). In this study, women without children had higher levels of self-objectification than mothers of young, and school-aged children. This protective state of motherhood could be due to the reduced pressure on women to find a partner, and have children, both of which are associated with sexual objectification (Brock et al., 2020). A recent systematic review of motherhood and objectification revealed that, across 18 studies, objectification by self and others was evident among mothers, but none of these studies compared women with and without children (Donati Beech et al., 2020). Research conducted among dyads of pregnant women and their partners found that when women reported feeling humanized by their partners, they had lower levels of objectification (Brock et al., 2020). Those authors suggested that pregnancy (and we extend this to the postpartum period) may be a unique time in the lives of women where “the tenets of objectification theory simply do not apply” (p. 10). Finally, in the case of dietary restraint, we suggest that women whose youngest child is aged 0 to 5 years may be more likely to be following intuitive eating, particularly if they are still breastfeeding, due to the known negative impact of dieting on breast milk supply. Lee et al. (2020) reported that 32% of women in the postpartum period (12 months post-birth) were classified as following intuitive eating, and these women experienced higher body satisfaction, less disordered eating, and lower depressive symptomatology.

BMI was not found to moderate the relationship between motherhood status and body shame. However, BMI was a significant predictor of body appreciation and body shame, such that women with higher BMI reported lower body appreciation and greater body shame. It seemed as though BMI was actually a stronger predictor of body image than our hypothesized motherhood status. Weight stigma is pervasive, and our research confirms that the body shame felt as a result of weight stigma is consistent across all women who have a higher BMI, irrespective of motherhood status. This increased body shame is likely to be the result of increased weight stigma, which has been found to be extremely harmful to individuals living in larger bodies (Tomiyama et al., 2018), and body shame is also known to prospectively predicts poorer health outcomes (Lamont, 2015). Given the considerable negative attention towards weight and obesity in media (Saguy et al., 2014), and the known negative physiological and psychological impacts of weight stigma (Wu and Berry, 2018), our research provides evidence that higher-weight women experience greater body shame and may need particular support to promote body appreciation in order to improve their health outcomes (Tylka and Homan, 2015).

Age was a significant negative predictor of self-objectification. This finding is consistent with previous research indicating that levels of objectification show a trend towards decreasing across the adult years in women (Tiggemann, 2004; Tiggemann and Lynch, 2001; Tiggemann and McCourt, 2013). It might be that, as they get older, women prioritize other thing, or that the impact of having a partner (and particularly a supportive one) reduces objectification over time (Brock et al., 2020). This is welcomed, as higher self-objectification has been linked to negative body image, disordered eating, depressed mood, and low sexual satisfaction (Tiggemann, 2011).

This is the first study, to our knowledge, to explore whether women with and without children have different levels of body appreciation, thin-ideal internalization, self-objectification, and body shame. The strengths of this study include the large community sample from diverse locations, as well as the use of standardized and validated instruments. Very little prior research has been conducted with large groups of women outside of college settings, and what has been done is often restricted to a local geographical area. However, a number of limitations in the current study should also be considered. In particular, the nature of participant recruitment—through the Body Image Movement social media—may have led to selection bias in our sample. In our sample, over 70% of women were following the Body Image Movement, and they may be doing this to seek support for their own body image, or conversely, to be body image advocates and champions in the community (Yager et al., 2020). Interestingly, in their sample of 738 women in the USA, Becker and Colleagues reported a higher mean Body Appreciation Score for their overall sample (25–86 years), indicating that women in the current study, despite having a body appreciation score above the mid-point on the scale, had lower levels of body appreciation relative to other samples. Women in our sample also still had higher body shame scores than in a study of university-aged women (Toole and Craighead, 2016) and lower levels of body appreciation than a community-sample of Canadian Women (Robbins and Reissing, 2018). Second, while we measured level of education as a proxy for level of income, and there were no differences between groups on this, it is possible that other demographic factors that were not measured might have influenced the results. For example, some research indicates that undergraduate women who are not in romantic relationships exhibit higher levels of self-objectification (Sanchez and Broccoli, 2008). We did not measure relationship status, and this could be important to consider in relation to future research on self-objectification across the lifespan and motherhood.

We recommend that future research continue to explore body image among adult women with larger, generalizable samples around the world. It might be possible for body dissatisfaction and body appreciation to co-exist in mothers, as found among adult women (Tiggemann and McCourt, 2013), and this should be investigated. As mothers play a key part in role modelling body attitudes (Damiano et al., 2019; Handford et al., 2018), and creating the home context in relation to food and physical activity (Lease et al., 2016), it is important that we also determine the potential impact of mothers’ body image on pre-school children.

Our findings also have potential implications for the development of intervention programs and resources for three key profiles of adult women, with and without children. While the mothers were not significantly worse off in terms of their body image and dieting attitudes and behaviors, compared to the women without children, intervention programs to build mothers’ body image are therefore still justified, particularly due to the critical role of mothers on their child’s body image and eating behavior (Hart et al., 2016). When mothers have a more positive body image, they are better able to role model this to their children (Damiano et al., 2019), and this is key to disrupting intergenerational transmission of body image concerns. The second cohort of adult women in need of intervention programs are those at higher weights. Inclusive programs that aim to improve body appreciation and reduce body shame for women at higher weights are needed in order to reduce the impact of weight stigma, and facilitate more positive health behaviors due to the known influence of body appreciation in improving physical activity, diet, and weight reduction or maintenance over time (Andrew et al., 2016; Bucchianeri et al., 2013; Neumark-Sztainer et al., 2006; Tylka and Homan, 2015). Finally, women without children might benefit from programs that aim to impact on self-objectification. While these interventions might differ in terms of the look and feel, and the format and practicalities of how they are presented and implemented, it is likely that self compassion, appreciation of body functionality, and mindful movement and intuitive eating approaches would be similarly helpful for all three of these intervention programs (Albertson et al., 2015; Alleva et al., 2015; Beintner et al., 2019; Seekis et al., 2020).

## Conclusion

Our research indicates that all women are vulnerable to body image concerns, but women with pre-school-aged children (0–5 years) had lower levels of body shame, dietary restraint, and self-objectification. There is a need for resources to support adult women in developing body appreciation, and reducing body shame, and these might be tailored based on individual variables such as BMI and practical stages of life such as motherhood in order to better meet the needs of targeted groups. Creating programs that improve mothers’ body image in particular, may also have the benefit of enhancing the home body image environment that might reduce the risk of the next generation of children developing body image concerns to the extent that we see today.
